# Acute Abdominal Pain and Radiological Pneumoperitoneum - Always an Indication for Laparotomy?

**DOI:** 10.4021/jocmr929w

**Published:** 2013-02-25

**Authors:** Christopher Buckle, Christopher Holdridge, Tina Xu, Falah Akhwais, Aaditya Sinha, Sudeendra Doddi, Prakash Sinha

**Affiliations:** a Department of General Surgery, Princess Royal University Hospital, Farnborough Common, Orpington, Kent, UK; b Charles University in Prague, Faculty of Medicine in Hradec Kralove, PO BOX 38, Simkova 870, Hradec Kralove 1, 500 38, Czech Republic

**Keywords:** Pneumoperitoneum, Pneumatosis intestinalis, Laparotomy, Conservative management

## Abstract

Pneumoperitoneum in the presence of acute abdominal pain is well recognised as an indication for laparotomy. We present a case of acute abdominal pain in the presence of an incidental pneumoperitoneum secondary to the rupture of pneumatosis intestinalis. We will discuss the importance of clinical context in the diagnosis and management of pneumoperitoneum and pneumatosis intestinalis.

## Introduction

Pneumatosis intestinalis (PI) is when there is bowel gas in the wall of the intestine. It could be primary when it is asymptomatic, or secondary to serious conditions such as bowel ischaemia.Pneumoperitoneum secondary to rupture of primary PI can be managed conservatively. Hence one should be aware of this rare but important condition.

## Case Report

A 91-year-old female presented to the accident and emergency department with a 12 hour history of severe right upper quadrant pain. She reported several similar previous episodes over the last year. On examination she was found to be tender in the right upper quadrant with localised guarding, but no evidence of generalised peritonism. Her initial blood tests demonstrated raised inflammatory markers and normal arterial blood gas and lactate measurements. An initial working diagnosis of acute calculus cholecystitis was formulated and management instigated.

**Figure 1:**
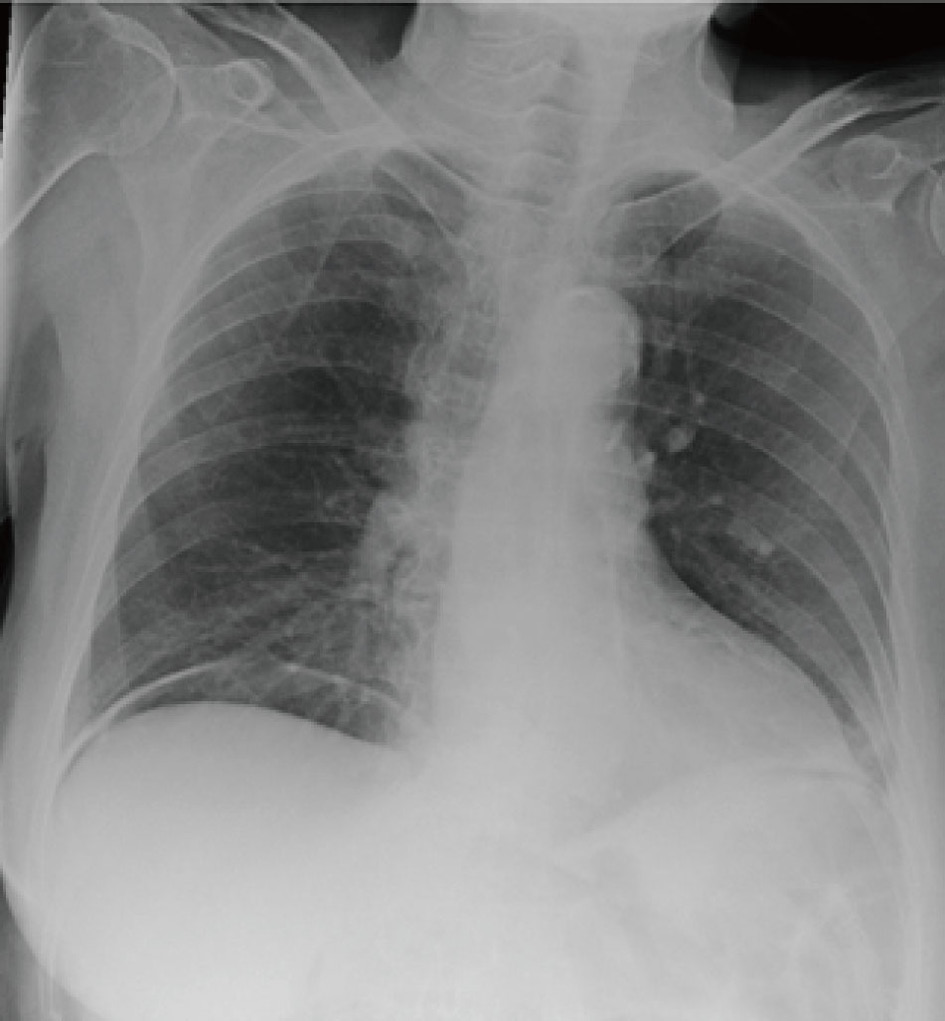
Erect chest x-ray demonstrating free gas under the right and left hemi-diaphragms.

**Figure 2:**
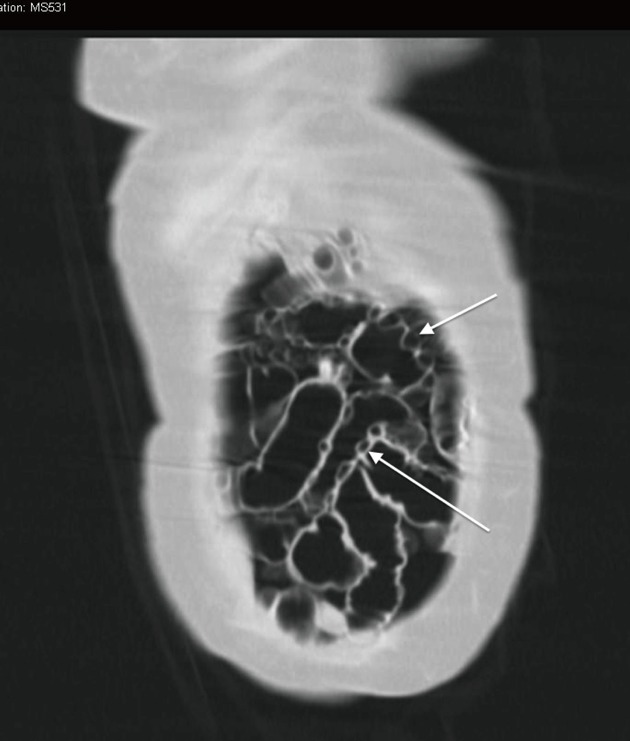
Coronal section of an abdominal computerised tomography demonstrating extensive intramural gas (white arrows).

Subsequently a routine erect chest x-ray was performed that demonstrated a large volume of free gas under the right hemi-diaphragm (Fig. 1). In view of this new finding the provisional diagnosis was revised to include bowel perforation. Computerised tomography (CT) of the abdomen was urgently performed and demonstrated typical features of cholecystitis. In addition the finding of pneumatosis intestinalis (PI) with large amounts of free intraperitoneal air was noted (Fig. 2). Importantly previous CT images were available from investigations for chronic lower abdominal pain and bloating. These demonstrated the pre existence of uncomplicated PI. The large volume of free intra peritoneal gas in the absence of generalised peritonism, abnormal blood gases and lactate suggested that the pneumoperitoneum was benign in nature and likely secondary to the rupture of PI. Treatment for acute cholecystitis was initiated and the patient carefully observed. She made an uneventful recovery, was discharged and underwent a laparoscopic cholecystectomy at a later date.

## Discussion

Pneumatosis intestinalis (PI) is defined as the presence of gas within the bowel wall (Fig. 2). It was first described by Du Vernoi (1783) and has subsequently been described using a variety of terms including pneumatosis coli, pneumatosis cystoides intestinalis and intramural gas. PI is not a diagnosis but rather a radiological or pathological finding and should therefore be interpreted within the clinical context it is found [1]. Its incidence is not well described, as it is often asymptomatic and therefore not clinically recognised [1]. PI in adults is typically found after the 5th decade and can be thought of as primary (idiopathic) (15%) or secondary (85%) [2]. Secondary PI and can be associated with a wide variety of conditions (Table 1), including those associated with significant mortality such as intestinal perforation and ischaemia [3]. Primary PI is often asymptomatic but can present with abdominal pain, distension and altered bowel habit, as in this case. A diagnosis may be made on plain x-ray, contrast studies, computerised tomography and endoscopy. Case reports have documented the formation of a pneumoperitoneum due to rupture of intra mural gas through the serosa into the peritoneal space, although the incidence of this is not well described [4].

**Table 1:** Conditions Associated With Pneumatosis Intestinalis [3]

Etiology	Examples
Intra abdominal catastrophe	Intestinal ischaemia, perforation or obstruction
Mucosal disruption	Peptic ulcer disease, inflammatory bowel disease
Infections	
Pulmonary disorders	Chronic obstructive pulmonary disease, asthma, mechanical ventilation
Iatrogenic	Endoscopy
Reduced intestinal motility	Diabetes, pseudo obstruction
Immunological compromise	Steroids, chemotherapy

Three major pathogenic theories have been proposed for the formation of PI: mechanical, bacterial and biomechanical [3]. The mechanical theory postulates that intra luminal gas enters the bowel wall due to mechanical forces via mucosal weaknesses. This would explain the presence of intra mural gas in the context of conditions such as obstruction, trauma, ischemia and inflammation. The bacterial theory postulates that gas-forming bacteria (such as clostridium perfringens) enter the bowel wall via mucosal breaks. The biomechanical theory suggests that bacteria within the lumen produce excessive amounts of hydrogen gas that under pressure pass through mucosal weaknesses into the bowel wall. In this case no obvious aetiological factor could be identified.

The management of PI is entirely dependent on the aetiology responsible for its presence and the severity of symptoms. In the case of incidental discovery and benign presentation conservative management is indicated. Treatment of symptomatic PI in the absence of an acute intra abdominal emergency includes the use of antibiotics, elemental diet and oxygen therapy [2]. In the case of an acute intra abdominal catastrophe such patients should immediately be considered for surgical management.

Our patient presented a more problematic clinical picture in which an acute surgical abdomen was complicated with the presence of a pneumoperitoneum and pre existing PI. This presents as diagnostic conundrum in which the surgeon must evaluate the suspected pathology of both the pneumoperitoneum and the abdominal pain. Knechtle suggested several clinical features that may predict the need for surgical exploration in cases of PI [5]. These include a clinical history and examination in keeping with an acute intra abdominal process; metabolic acidosis including elevated lactate measurements and an elevated amylase. Our patient although presenting with acute abdominal pain had only localised tenderness and did not have any features of metabolic disturbance. For these reasons the avoidance of laparotomy can be justified.

### Conclusion

Pneumatosis intestinalis is a clinical finding that can be associated with a pneumoperitoneum. It is a condition that can be incidental or indicate significant intra abdominal pathology. In this case an emergency laparotomy was avoided despite the presence of significant intra peritoneal gas in a patient with acute abdominal pain as the patient did not have features of peritonism or metabolic disturbance. This decision was further strengthened in view of the historical nature of the PI. For this reason this case highlights the importance of the application of sound clinical judgement in the context of the patient in front of you, not simply an isolated image presented to you.
